# Impact of Sirt1 on mammalian aging

**DOI:** 10.18632/aging.100156

**Published:** 2010-06-12

**Authors:** Daniel Herranz, Manuel Serrano

**Affiliations:** Spanish National Cancer Research Center (CNIO), Madrid E-28029, Spain

**Keywords:** Herranz D et al. Sirt1 improves healthy ageing and protects from metabolic syndrome-associated cancer. Nat Commun 2010, 1:3.

Studies in yeast, flies and worms have indicated that overexpression of the protein deacetylase Sir2 increases longevity [1]. Mammals possess 7 paralogs of the Sir2 gene, being Sirt1 the most similar one closest to Sir2 [1]. In our recent work [2], we have used a genetic approach to address the effect of Sirt1 on mammalian ageing. In particular, we have generated two independent lines of transgenic mice that globally overexpress Sirt1 (≈3-fold) under the control of its own regulatory elements (Sirt1-tg mice). Previously, we had shown that these Sirt1-tg mice are protected from the metabolic damage associated to high-fat diet (HFD) through the inhibition of NFκB inflammatory pathway and the activation of PGC1α antioxidant response [3]. The potent beneficial effects of Sirt1 in protecting from metabolic syndrome and its associated pathologies, such as diabetes and fatty liver, have consistently emerged in a variety of mouse models as one of the main physiological activities of Sirt1 [3-7]. We now report the ageing and longevity of Sirt1-tg mice [2].

We have observed that old Sirt1-tg mice show a better health during aging compared to their wild-type littermates. In particular, old Sirt1-tg mice are partially protected from diabetes, osteoporosis and cancer. However, these beneficial effects on health are not potent enough to increase lifespan and the survival curves of Sirt-tg and wild-type mice are indistinguishable. Failure to extend longevity may simply reflect the fact that Sirt1 overexpression in Sirt1-tg mice is not sufficiently high, or it may indicate that Sirt1 does not protect from all aging-associated pathologies. In this regard, Sirt1-tg mice showed a significant decrease in the incidence of carcinomas and sarcomas, but their incidence of lymphomas was comparable to wild-type mice. Of note, lymphomas are the most abundant cancer type in the strain of mice used and are a probable cause of death [8]; while, in general, carcinomas and sarcomas in old mice are at an incipient stage and mice presumably die of other aging-associated pathologies. Similarly, it is conceivable that other pathologies not studied in our work, such as cardiovascular or renal failure, could be insensitive to Sirt1 or may require higher levels of Sirt1 to be ameliorated. Finally, other Sir2 paralogs present in the mammalian genome may also contribute with important anti-aging functions and together recapitulate the longevity effect of Sir2 in other model organisms.

**Figure 1.**
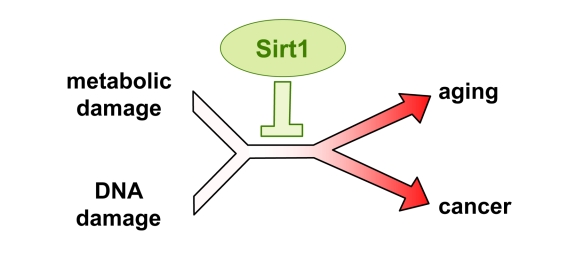
Sirt1 protects from damage and improves healthspan.

Another aspect of novelty in our work is the employment of a new model of diet-promoted liver cancer and the demonstration that Sirt1 is a potent protector of this type of liver carcinogenesis. In particular, we performed a protocol based on the injection of the hepatocarcinogen ditehylnitrosamine (DEN) followed by high-fat diet (HFD) feeding. Independently, other investigators have recently demonstrated that HFD promotes liver cancer through the induction of systemic inflammation  [9]. In the case of Sirt1, we show that it protects dramatically from this type of carcinogenesis not only by diminishing the inflammatory response associated to HFD, but also by protecting from the initial acute DNA damage triggered by DEN [2]. The latter observation is in concordance with previous *in vitro* reports that demonstrated a role for Sirt1 in DNA repair  [10, 11]. Together, and despite the lack of effect on longevity, our results demonstrate a beneficial role for Sirt1 in liver damage, metabolic syndrome-associated liver cancer, and in a variety of aging-associated pathologies, such as spontaneous carcinomas and sarcomas, diabetes and osteoporosis  [2] (Figure 1).
